# Supplementary material for: Bio-Guided Isolation of SARS-CoV-2 Main Protease Inhibitors from Medicinal Plants: In Vitro Assay and Molecular Dynamics
Source: Plants (Basel). 2022 Jul 24;11(15):1914. doi: 10.3390/plants11151914 (PMC9332707; doi:10.3390/plants11151914)
Supplement: Supplementary file 1 [file plants-11-01914-s001.zip › plants-1796763-supplementary.pdf]

Supplementary Materials

Bio-guided Isolation of SARS-COV-2 Main Protease Inhibitors from Medicinal Plants: *In vitro* Assay and Molecular Dynamics

Hossam M. Abdallah [1,*,†], Ali M. El-Halawany [2], Khaled M. Darwish[3], Mardi M. Algandaby[4], Gamal A. Mohamed[1], Sabrin R. M. Ibrahim[5,6], Abdulrahman E. Koshak[1], Sameh S. Elhady[1], Sana A. Fadil[1], Ali A. Alqarni[1], Ashraf B. Abdel-Naim[7] and Mahmoud A. Elfaky[1]

[1] Department of Natural Products and Alternative Medicine, Faculty of Pharmacy, King Abdulaziz University, Jeddah 21589, Saudi Arabia gahussein@kau.edu.sa (G.A.M.); aekoshak@kau.edu.sa (A.E.K.); ssahmed@kau.edu.sa (S.S.E.); Safadil@kau.edu.sa (S.A.F.); ali.abdullah.10@hotmail.com (A.A.A.); melfaky@kau.edu.sa (M.A.E.)
[2] Department of Pharmacognosy, Faculty of Pharmacy, Cairo University, Cairo 11562, Egypt; ali.elhalawany@pharma.cu.edu.eg
[3] Department of Medicinal Chemistry, Faculty of Pharmacy, Suez Canal University, Ismailia 41522, Egypt; khaled_darwish@pharm.suez.edu.eg
[4] Department of Biological Sciences, Faculty of Science, King Abdulaziz University, Jeddah 21589, Saudi Arabia; malgandaby@kau.edu.sa
[5] Preparatory Year Program, Department of Chemistry, Batterjee Medical College, Jeddah 21442, Saudi Arabia; sabrin.ibrahim@bmc.edu.sa
[6] Department of Pharmacognosy, Faculty of Pharmacy, Assiut University, Assiut 71526, Egypt
[7] Department of Pharmacology, Faculty of Pharmacy, King Abdulaziz University, Jeddah 21589, Saudi Arabia

* Correspondence: hmafifi@kau.edu.sa, Tel.: +966-544-733-110

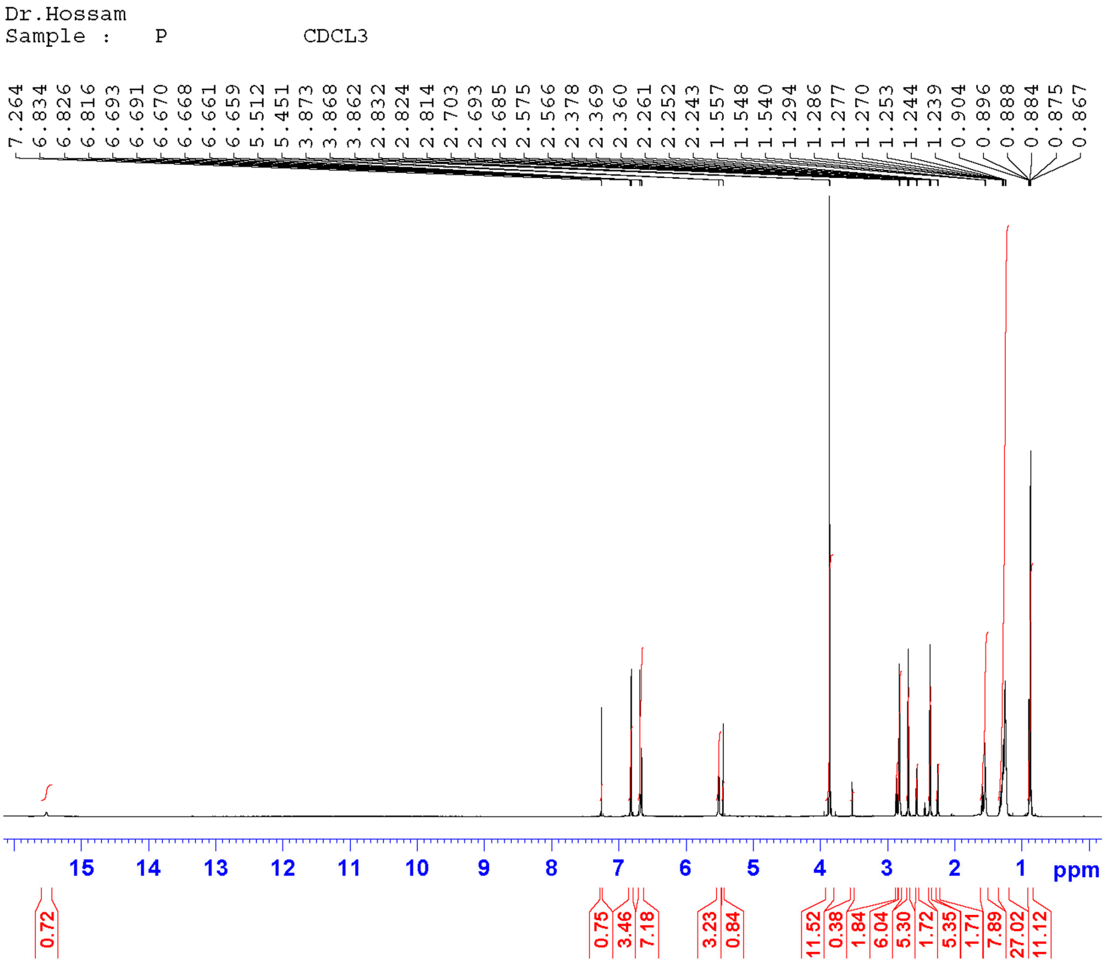

Figure S1. ^{1}HNMR spectra of 6-paradol.

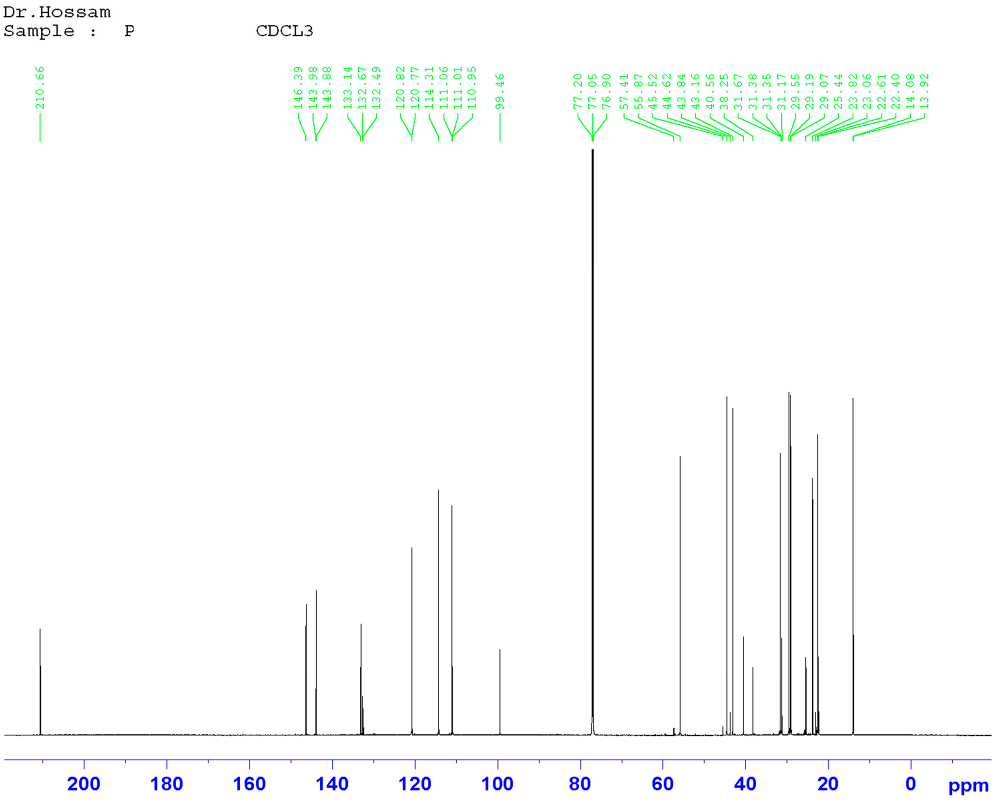

Figure S2. ^{13}CNMR spectra of 6-paradol.

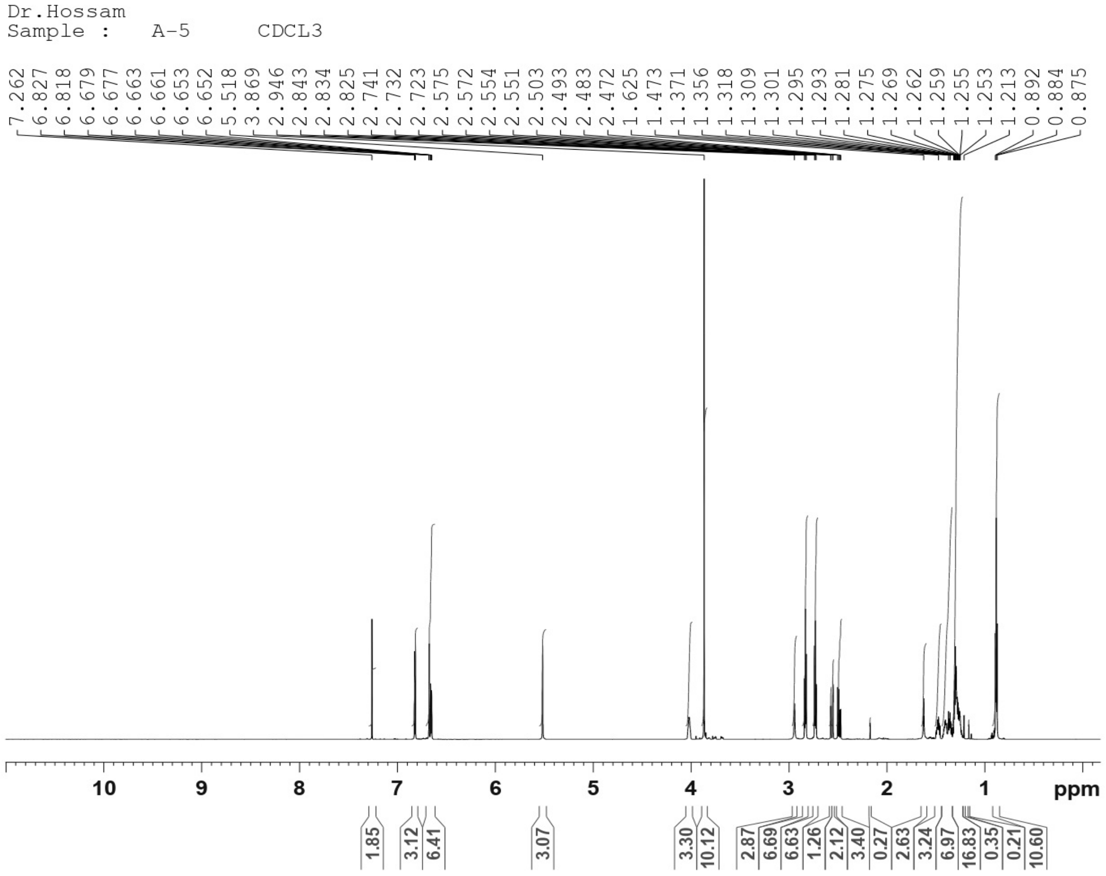

Figure S3. ^{1}HNMR spectra of 6-gingerol.

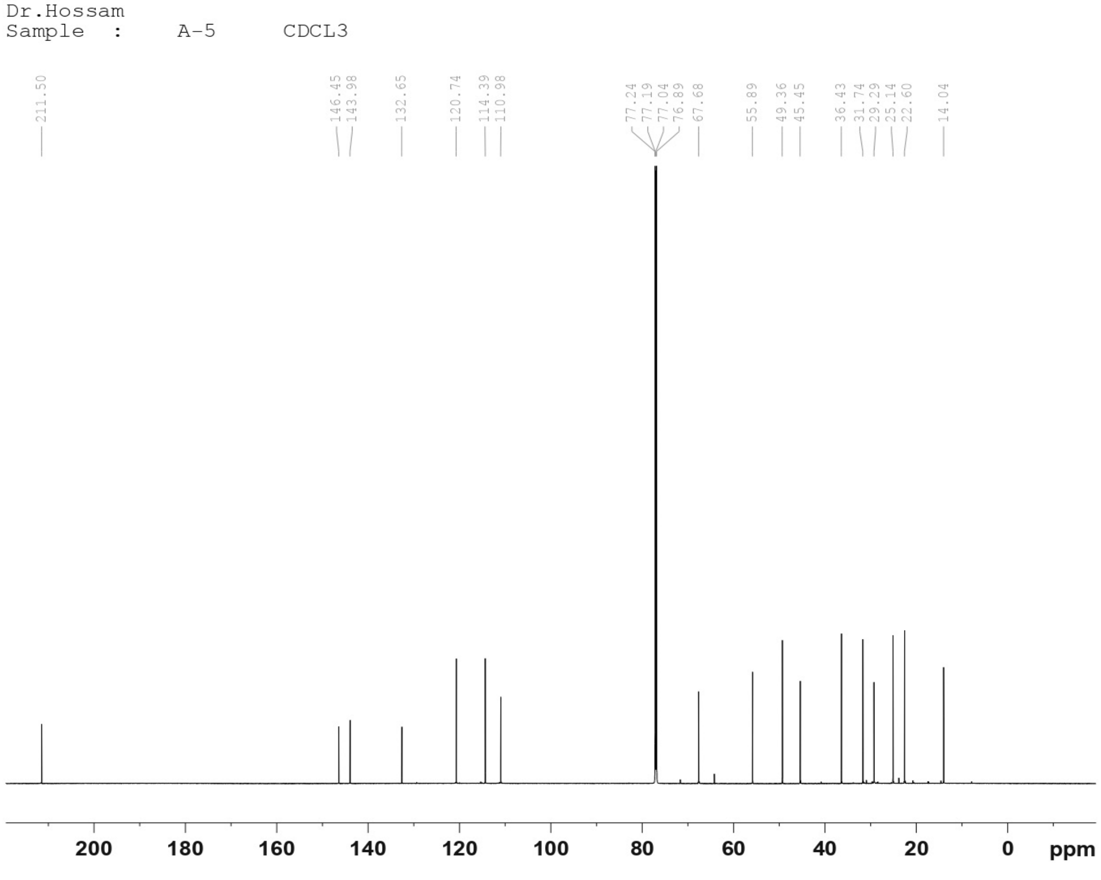

Figure S4. ^{13}CNMR spectra 6-gingerol.

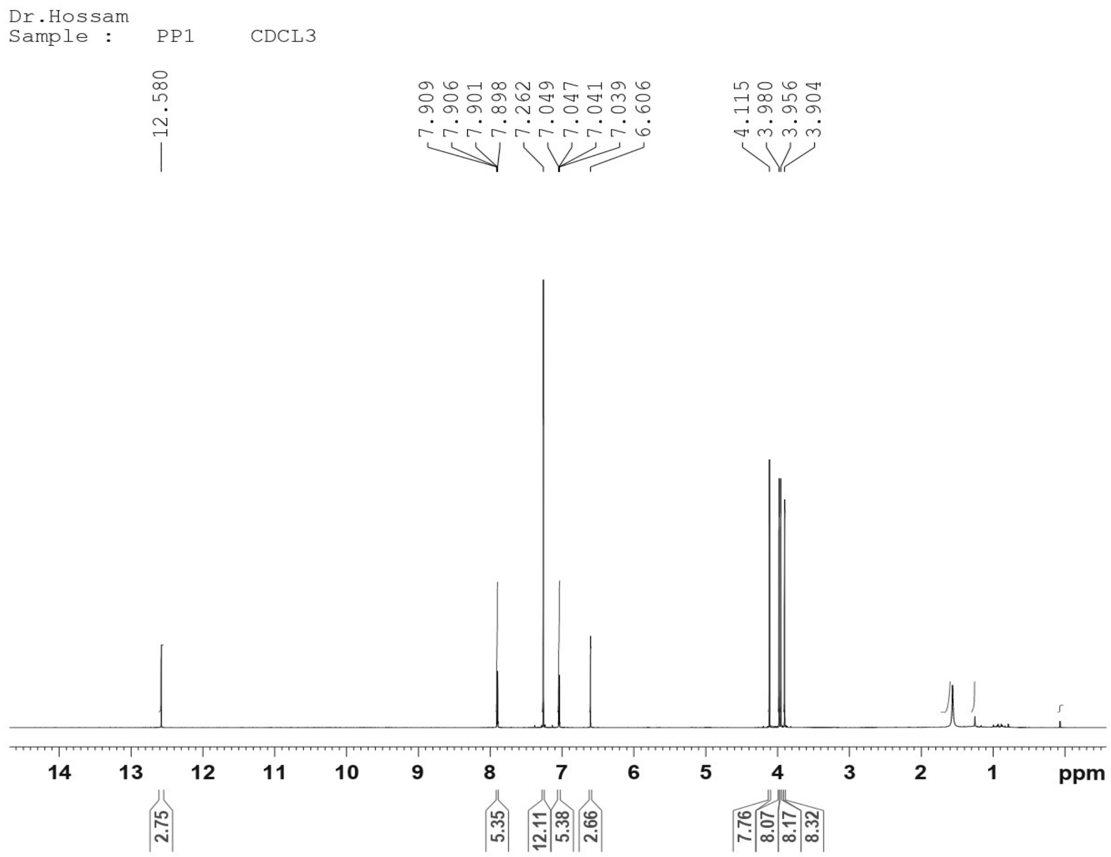

Figure S5. ^{1}HNMR spectra of gardenin B.

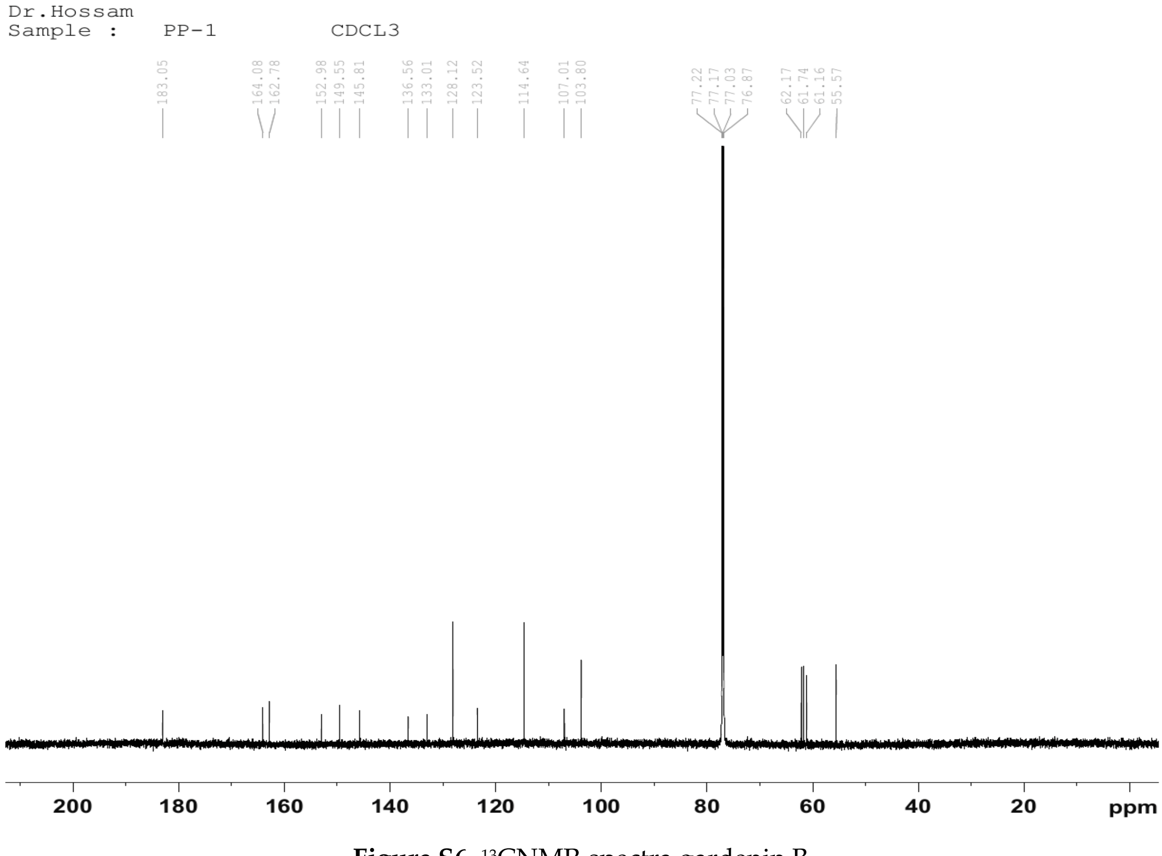

Figure S6. ^{13}CNMR spectra gardenin B.

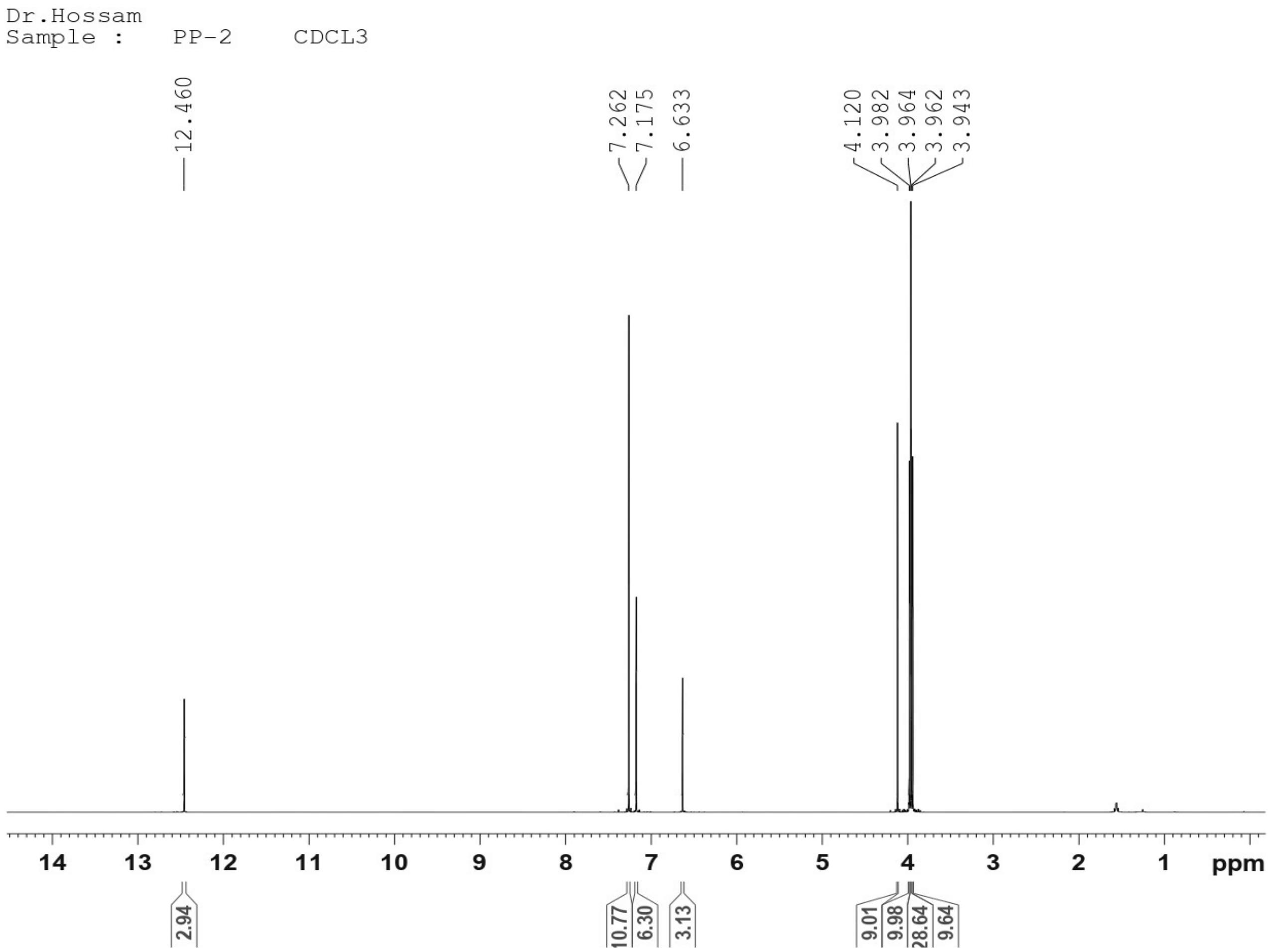

Figure S7. ^{1}HNMR spectra of gardenin A.

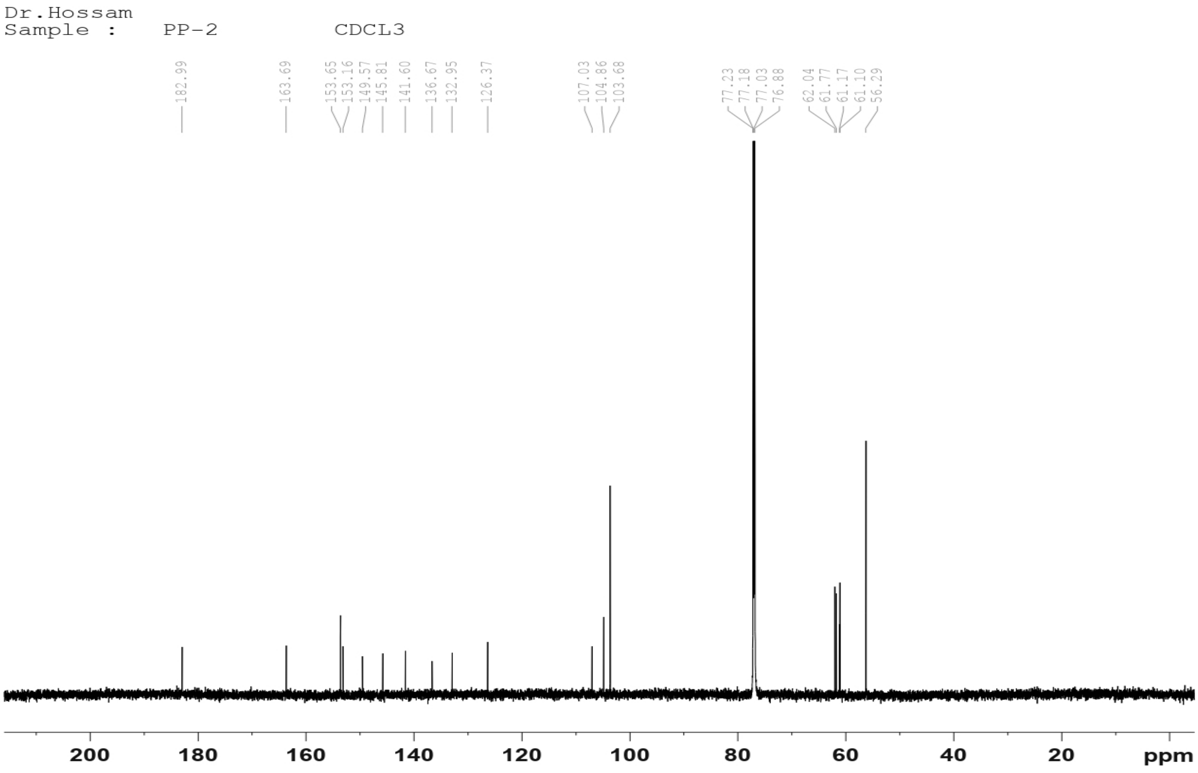

Figure S8. ^{13}CNMR spectra gardenin A.

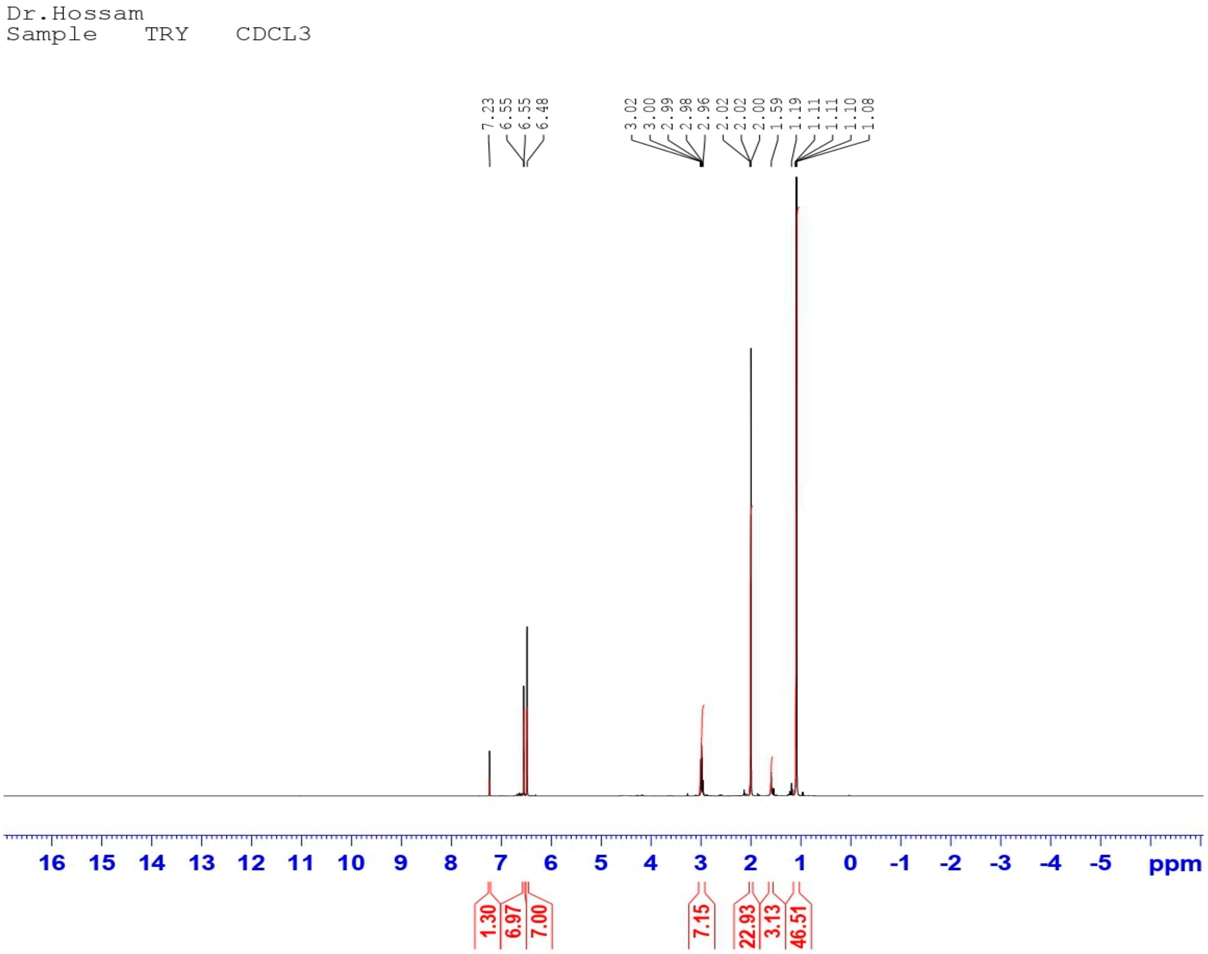

Figure S9. ^{1}HNMR spectra of thymoquinone.

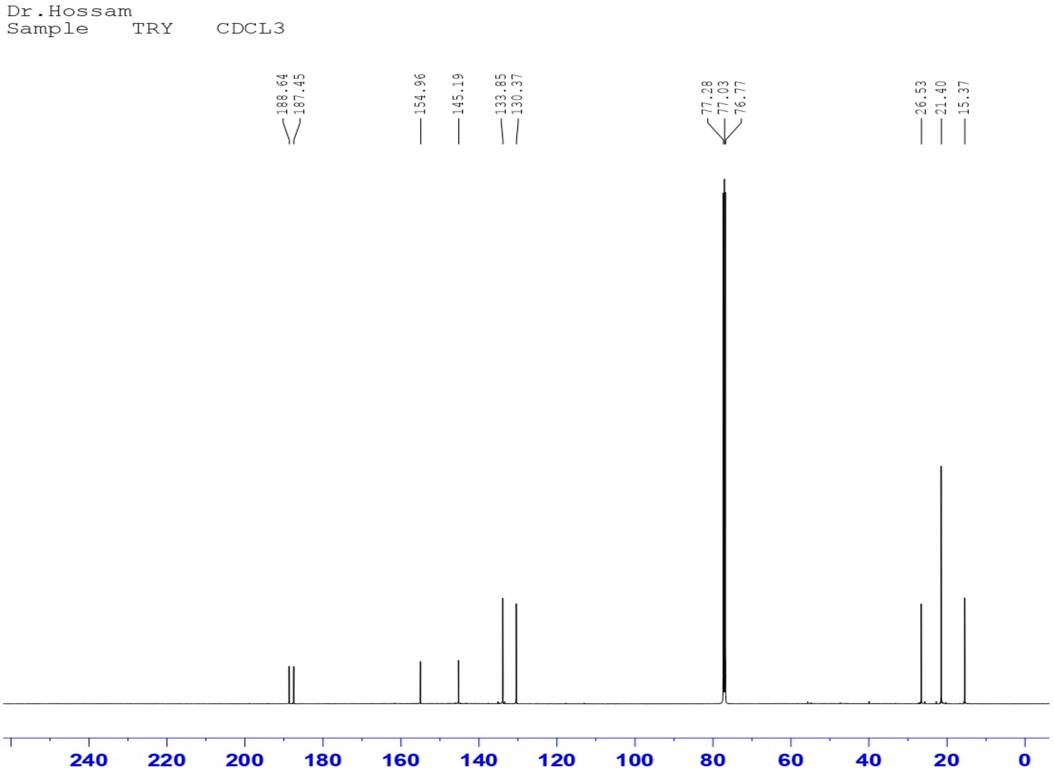

Figure S10. ^{13}CNMR spectra thymoquinone.

(**A**) (**B**)

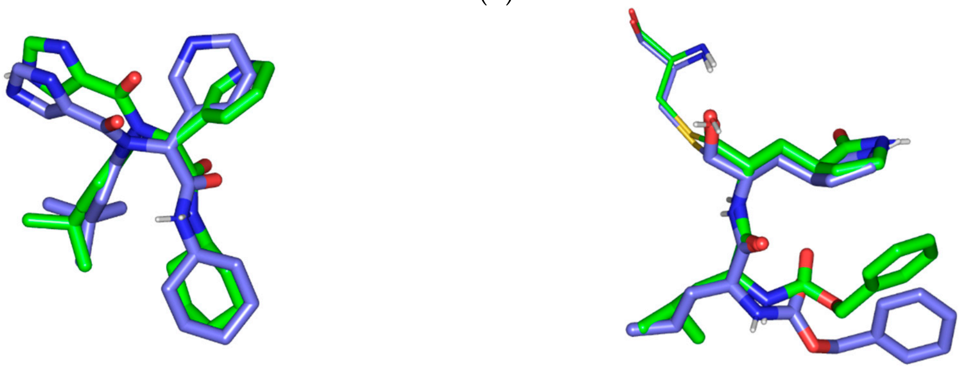

Figure S11. Superimposing the co-crystallized (blue sticks) and redocked (green sticks) ligands. (**A**) non-covalent X77 (PDB: 6W63); (**B**) covalent GC376 (PDB: 7CBT) forming hemi-thioacetal adduct with Cys145 (lines) for validating the adopted docking protocols.

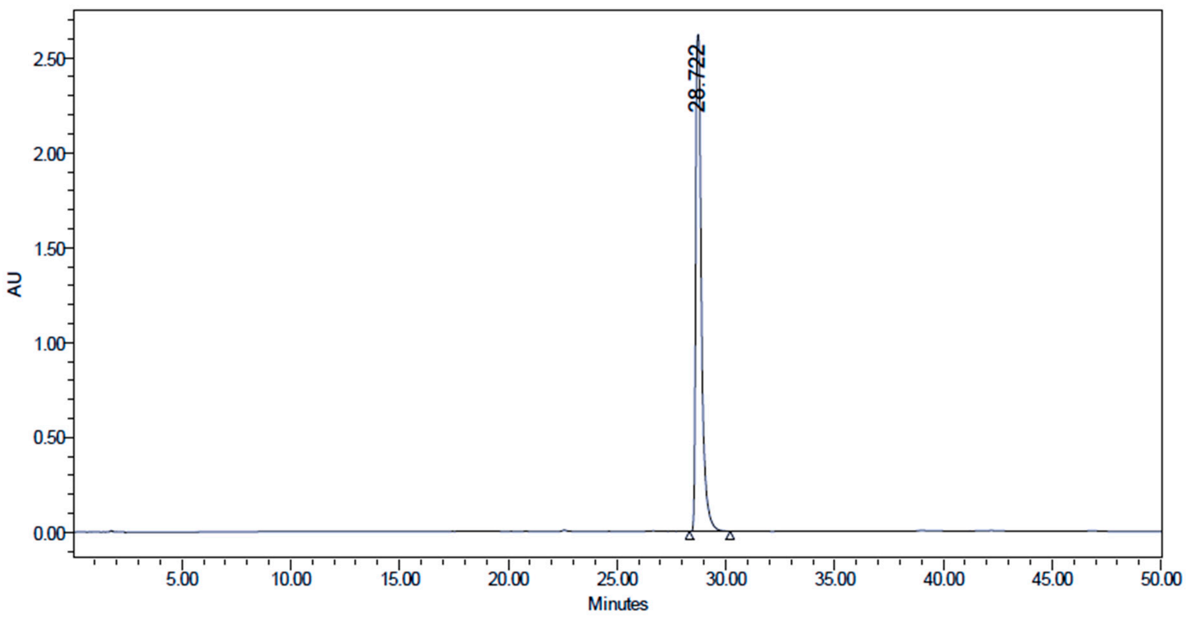

Figure S12. Thymoquinone HPLC chromatogram
HPLC was carried out on Waters W2690/5 system equipped with 996 PAD and Empower 3 Software Build 3471 SPs Installed: Feature Release 3. The column used was Kromasil (Sweden) 4.6X150mmX5um. Thymoquinone purity assessment was carried out using mobile phase A (phosphate buffer 20mM Ph 2.) and B; CH_3CN at 1ml/min flow rate according to the following gradient: 0 min. 95% A, 2 in 95% A, 40 min 20% A43min 20% A. Spectra were recorded at 253nm. Using the previous condition, the compound purity was found to be 99.5%

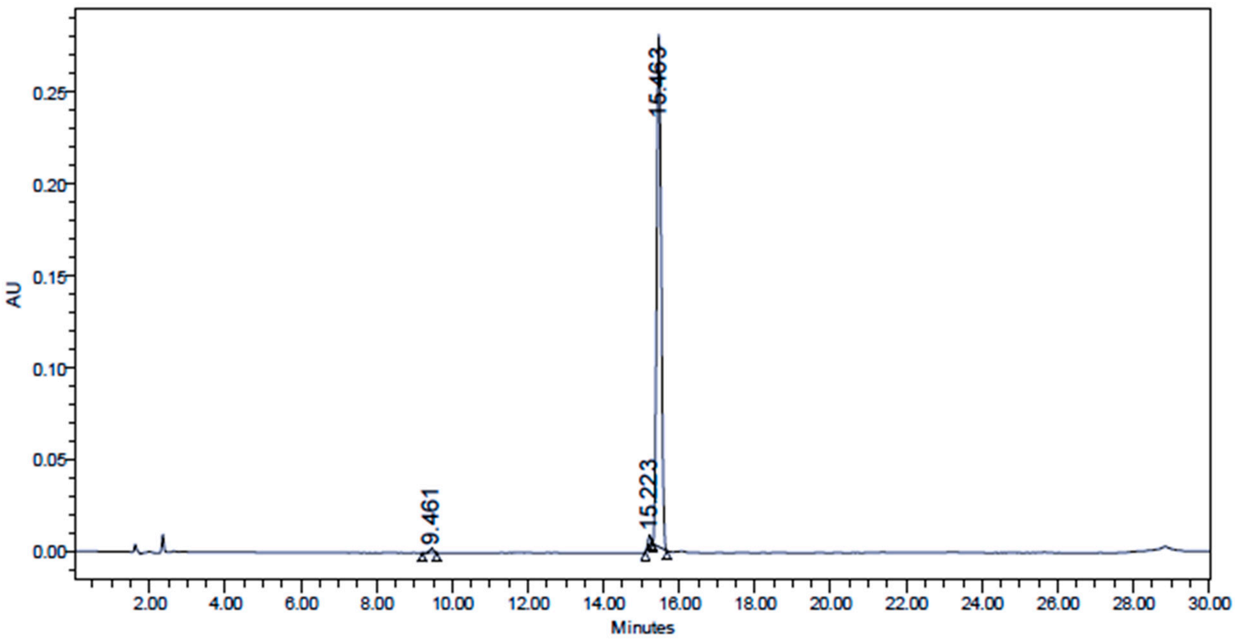

Figure S13. 6-Gingerol HPLC chromatogram
HPLC was carried out on Waters W2690/5 system equipped with 996 PAD and Empower 3 Software Build 3471 SPs Installed: Feature Release 3. The column used was Kromasil (Sweden) 4.6X150mmX5um. Gingerol purity was determined using Inertsil C18 4.6X150mmX5um and mobile phase consisting of 0.1% orthophosphoric acid (A) and CH_3CN (B) gradient elution was as follows; 0 min. 80%A, 2 min 80% A, 20 min 20% A25 min 20% A. elution was carried out at flow rate of 1 ml/ min and spectra were recorded at 280 nm

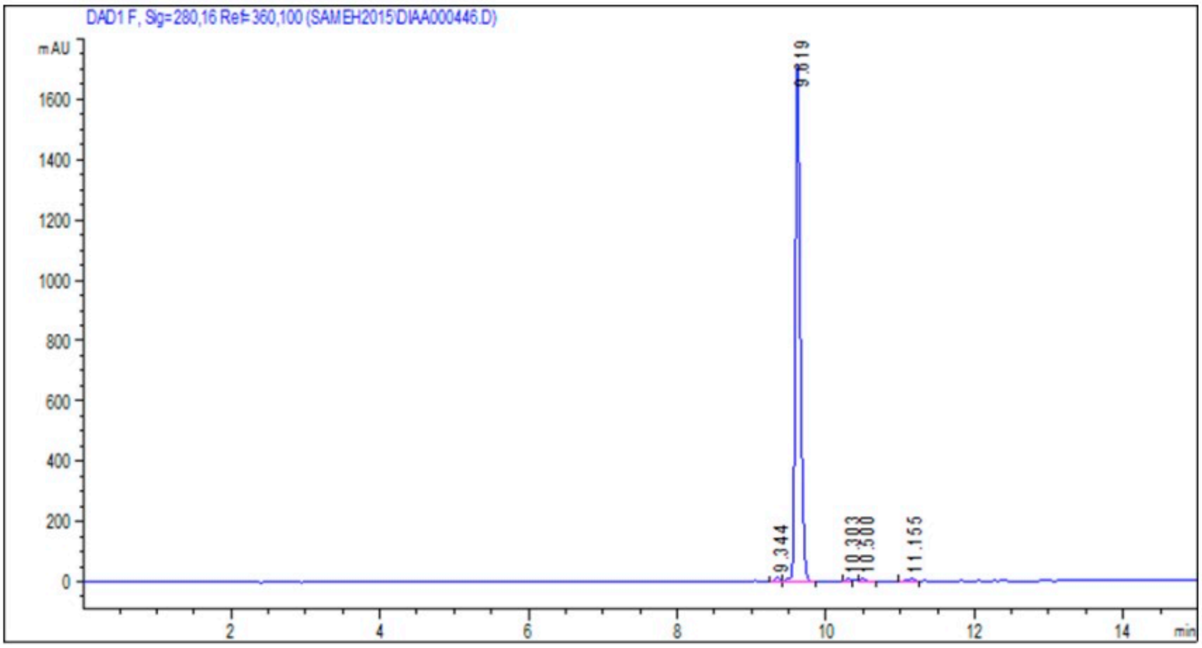

Figure S14. 6-Paradol HPLC chromatogram
HPLC was carried out on Agilent 1200 infinity instrument equipped with automatic injector and DAD detector. Paradol was chromatographed gradiently using a mobile phase A (acetonitrile) and B (0.1% TFA). The gradient elution program was: 35 % A (0-2 min), 35 - 60 % A (2-10 min), 60 - 100 % A (10-12 min) and 100 - 35 % A (12-16 min). The flow rate was 1.0 mL/min and the peaks were monitored 280 nm, respectively. Purity of paradol was 97% at these conditions

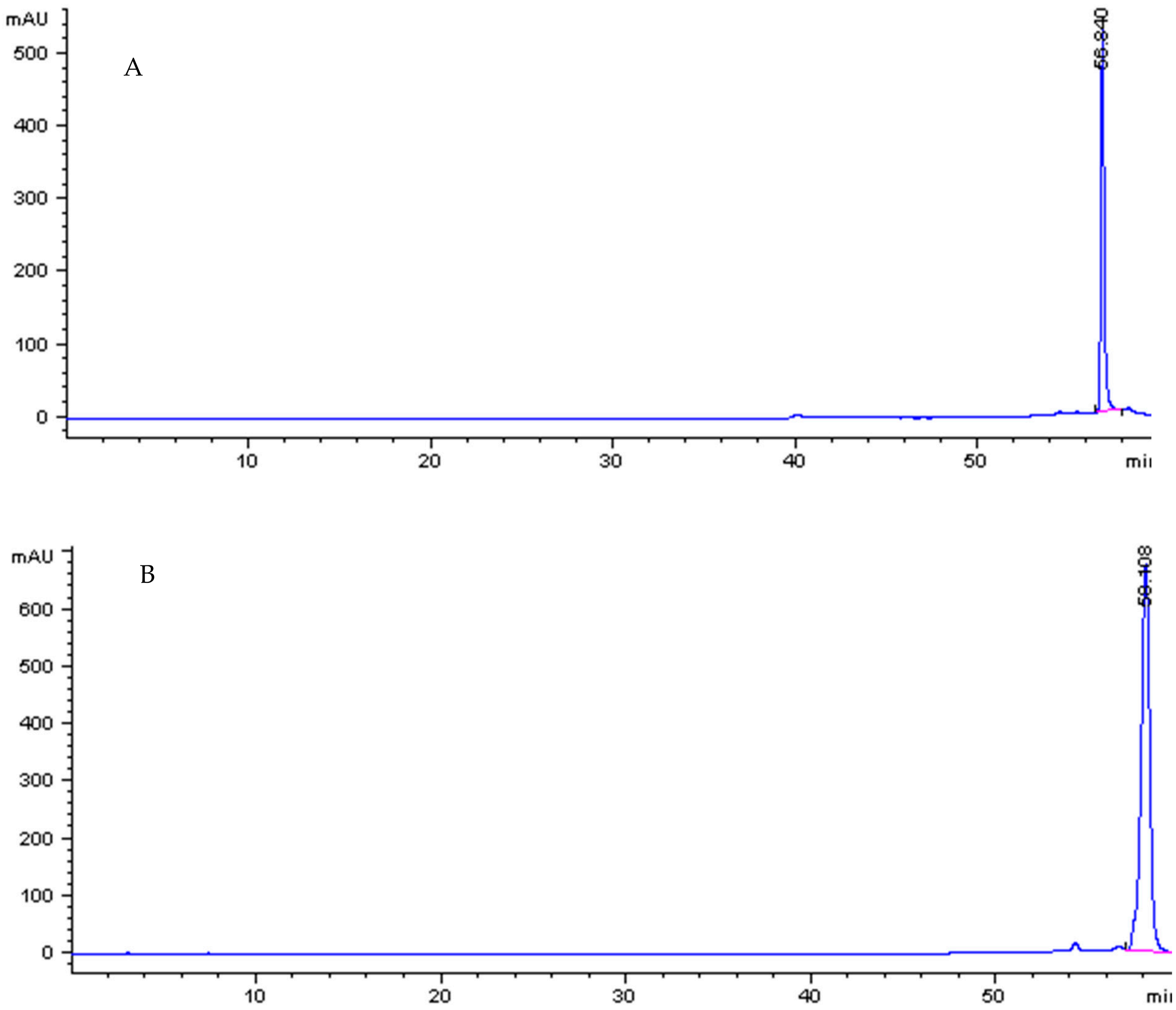

Figure S15. Gardenin A (A) and Gardenin B (B) HPLC chromatogram

HPLC system consisted of an Agilent 1200 system, a solvent delivery module, a quaternary pump, an autosampler, a diode-array detector (DAD), and a column compartment (Agilent Zorbax Extend-C18 column (250 mm length × 4.6 mm, i.d, μm) using Mobile phase: Acetonitril (solvent A) & 0.1% formic acid (solvent B), The gradient elution program was: 20-8% A (0-3 min), 8-42% A (3-45 min), 42-64% A (45-54 min), 64-20% A (54-60 min), 20% A (60-65 min). The flow rate was 1.0 mL/min and the peaks were monitored 280 nm, respectively. Purity of Gardenin A (A) and Gardenin B (B) were 97% at these conditions
